# Deep Proteome Coverage of Microglia Using a Streamlined Data-Independent Acquisition-Based Proteomic Workflow: Method Consideration for a Phenotypically Diverse Cell Type

**DOI:** 10.3390/proteomes12040035

**Published:** 2024-11-27

**Authors:** Jessica Wohlfahrt, Jennifer Guergues, Stanley M. Stevens

**Affiliations:** Department of Molecular Biosciences, University of South Florida, Tampa, FL 33620, USA

**Keywords:** microglia, DIA-PASEF, DDA library generation, deep proteomics, DIA methods development

## Abstract

As the primary innate immune cells of the brain, microglia play a key role in various homeostatic and disease-related processes. To carry out their numerous functions, microglia adopt a wide range of phenotypic states. The proteomic landscape represents a more accurate molecular representation of these phenotypes; however, microglia present unique challenges for proteomic analysis. This study implemented a streamlined liquid- and gas-phase fractionation method with data-dependent acquisition (DDA) and parallel accumulation–serial fragmentation (PASEF) analysis on a TIMS-TOF instrument to compile a comprehensive protein library obtained from adult-derived, immortalized mouse microglia with low starting material (10 µg). The empirical library consisted of 9140 microglial proteins and was utilized to identify an average of 7264 proteins/run from single-shot, data-independent acquisition (DIA)-based analysis microglial cell lysate digest (200 ng). Additionally, a predicted library facilitated the identification of 7519 average proteins/run from the same DIA data, revealing complementary coverage compared with the empirical library and collectively increasing coverage to approximately 8000 proteins. Importantly, several microglia-relevant pathways were uniquely identified with the empirical library approach. Overall, we report a simplified, reproducible approach to address the proteome complexity of microglia using low sample input and show the importance of library optimization for this phenotypically diverse cell type.

## 1. Introduction

Microglia are the resident immune cells of the central nervous system and are intimately involved in various processes of homeostatic maintenance as well as immune reactivity to pathophysiological conditions within the brain. In this regard, microglia can display a wide range of phenotypes to carry out important functions such as cell migration, phagocytosis, and the release of cytokines/chemokines. Given the importance of the proteome as a molecular signature that more accurately describes microglial phenotype, comprehensive proteome coverage can provide critical insight into the involvement of microglia in fundamental biological processes in the brain as well as their potential contribution in the pathogenesis and progression of neurodegenerative diseases. However, the phenotypic diversity of microglia, which ultimately allows them to carry out these important immune-related functions, also presents biological challenges related to the reproducible detection of all relevant proteins underlying the highly complex and context-dependent molecular profiles associated with microglial reactivity. Additionally, in terms of experimental challenges, the number of microglia and related protein amount may be limited (e.g., FACS-sorted microglia from a specific mouse brain region) and can hinder the identification of key proteins driving microglial phenotype in single-shot proteomics experiments.

Technological and informatic advancements have addressed some of these challenges, including proteome complexity, and have allowed for significant improvement in the overall depth of microglial proteome coverage. Specifically, previous studies from our lab as well as others have shown a steady improvement in microglial proteome coverage over time using various mass spectrometry-based proteomic approaches (Table 1). An early study from our lab in 2012 utilized strong cation exchange (SCX) offline HPLC fractionation and an LTQ Orbitrap XL to characterize a rat microglial cell line at a depth of coverage of 3006 total unique protein groups [1]. Work by Rangaraju et al. in 2018 identified 4133 proteins from acutely isolated adult-derived mouse microglia by employing electrostatic repulsion hydrophilic interaction chromatography (ERLIC) fractionation in combination with tandem mass tag (TMT)-based proteomic analysis on an Orbitrap Fusion instrument [2]. A recent study by Lloyd et al. in 2024 identified a total of 9633 proteins collectively from two different mouse-derived microglial cell models using deep offline high-pH reversed-phase (RP) HPLC fractionation in combination with LC-MS analysis on a Q Exactive Plus (Orbitrap) instrument [3]. While these types of comprehensive studies have facilitated the identification of a larger number of microglial proteins, significant progress toward deep proteome coverage in a single shot has also been accomplished. As an example, a study from our lab in 2020 used a segmented data-dependent acquisition (DDA) approach on a Q Exactive Plus (Orbitrap) instrument to collectively identify 5062 proteins from an adult-derived mouse microglial cell line (IMG cells) [4].

While almost all the aforementioned studies utilized a DDA approach, data-independent acquisition (DIA)-based analysis has become well recognized for its improved utility in deep, single-shot proteomic analysis, which includes advantages such as increased precursor detection sensitivity as well as low data missingness. However, a major limitation in DIA-based workflows has traditionally been the generation of a spectral library that is usually obtained by deep offline fractionation followed by LC-MS analysis. This process is both time- and labor-intensive and typically requires more protein sample input. Microglia, especially those derived from in vivo experiments, can be limited in cell count, necessitating conservation of starting material. Additionally, the proteins driving microglial phenotypic diversity might not be represented in the spectral library, preventing these features from being identified in subsequent DIA-based, single-shot analysis using the empirical spectral library. More recently, library-free DIA data analysis methods have been utilized and have helped circumvent some of the limitations of empirical spectral libraries [6]. Due to the diverse nature of microglia phenotypes, an in silico-predicted library employed in the library-free approaches could presumably identify proteins and related pathways that may not be represented in the empirical library but at the tradeoff of increased computational time when performing DIA data searches [7].

A previous paper from our lab introduced ion mobility (IM)-based gas-phase fractionation on a TIMS-TOF instrument [8] to generate an empirical library for DIA-based proteomic analysis using minimal sample starting material and significantly decreasing overall experimental hands-on time. Considering the limitations of microglial proteomic analysis and to strike a balance between proteome depth of coverage and throughput, we incorporated a facile solution-phase fractionation approach in combination with gas-phase IM fractionation, which aimed to improve the proteome depth of coverage of adult murine brain-derived immortalized microglial (IMG) cells that were used as the microglial cell model in this study [9,10]. Our straightforward approach was assessed in terms of fractionation reproducibility from low starting material of IMG cell lysate as well as utility of the generated empirical spectral library for subsequent single-shot, DIA-based analysis. Additionally, we evaluated the potential complementarity of the IMG-derived DIA output using both the empirical and predicted libraries with the overall goal of addressing the experimental challenges of this phenotypically diverse cell type.

## 2. Materials and Methods

### 2.1. Cell Culture and Collection

Immortalized mouse MicroGlial cells (IMG, Millipore, Burlington, MA, USA) were grown in T75 flasks containing DMEM supplemented with 100 U/mL penicillin, streptomycin, and 10% FBS. Flasks were incubated at 37 °C and 5% CO_2_. Once flasks had reached approximately 70% confluence, the cells were washed with ice-cold PBS and scrape-collected in additional PBS. Samples were then washed 2× more with ice-cold PBS with 300× *g* spin down at 4 °C for 7 min, and the supernatant was removed in between. After removing the supernatant from the final wash, cell pellets were flash-frozen using liquid nitrogen and stored at −80 °C until processing.

### 2.2. Sample Processing

IMG cells were processed with iST and iST peptide fractionation add-on (PreOmics, Planegg, Martinsried, DE, Germany), which generates three peptide fractions, according to manufacturer directions with only minor adjustments [11]. In brief, IMG cell pellets were lysed in 50 µL of iST Lyse reagent, heated at 95 °C for 10 min, and sonicated to shear DNA. At this point, protein amounts were normalized to 10 µg per sample and subsequently digested in a tube using 10 µL of iST Digest for 1 h at 37 °C and 500 rpm shaking. Digests were transferred into iST cartridges with 100 µL of iST Stop reagent and centrifuged at 3800× *g* for 2 min. Samples were washed with iST Wash 1 and 2, repeating the centrifugation in between. At this point, one set of *n* = 3 was fractionated, while the other was left unfractionated. For unfractionated samples, 100 µL was added to the column twice with a 2 min centrifugation step after the first addition and a 10 min centrifugation step after the second addition of sample. For the fractionated samples, the iST fractionation add-on buffers were used for elution. Fraction 1, 2, and 3 buffers were added sequentially to the sample cartridge and collected in separate tubes, respectively, by centrifuging at 1000× *g* for 1 min after each addition. All sets of eluted peptides were dried using a speed vac and stored at 4 °C until resuspension in 0.1% formic acid in LC-MS-grade water.

### 2.3. Mass Spectrometry Analysis for Empirical Library Generation

Digested microglial cell lysates were separated using an Aurora series Ultimate reversed-phase C18 column (25 cm × 75 µm i.d., 1.7 µm C18, IonOpticks, Collingwood, Victoria, Australia) at 50 °C in conjunction with a nanoElute UHPLC system (Bruker, Billerica, MA, USA) in line with a TIMS-TOF mass spectrometer (timsTOF Pro, Bruker). Mobile phases consisting of 0.1% formic acid in water (A) and 0.1% formic acid in acetonitrile (B) were utilized in a gradient of 2–25% B, which elutes the majority of the peptides off of the column. A following ramp up to 37–80% B was added at the end to clean and prepare the column, resulting in an increased total time. A gradient time of 120 min (160 min total) was used in order to generate the empirical library on all fractions as well as on a non-fractionated alignment sample, while gradient times of both 45 min (60 min total) and 90 min (120 min total) were used on non-fractionated samples to assess the comprehensive coverage in using this library compared with an in-silico predicted DIA spectral library.

For the purpose of generating the empirical fractionated library, DDA analysis with parallel accumulation–serial fragmentation (PASEF) was implemented using a 120 min (160 min total) gradient and two ion mobility (IM) fractions, 0.8–1.05 1/K_0_ [V·s/cm^2^] and 1.0–1.25 1/K_0_ [V·s/cm^2^] as described previously, to analyze the three iST peptide fractions in triplicate [8]. An alignment run was also included to allow for appropriate calibration of IM and retention time (RT) in a DIA analysis consisting of a single 120 min DDA file encompassing the entirety of the IM fractions into one overall range of 0.7–1.4 1/K_0_ [V·s/cm^2^]. DDA-PASEF method settings included an *m*/*z* range of 100–1700, with a ramp rate of 9.43 Hz, ramp time of 100 ms, an estimated total cycle time of 1.17 s with a target intensity of 15,000 and an intensity threshold of 2500, and an active exclusion list that releases after 0.4 min.

### 2.4. Generation of Spectral Libraries

DDA-PASEF data were analyzed by FragPipe (v. 19.1) with MSFragger (v. 3.7), IonQuant (v. 1.8.10), and Philosopher (v. 4.8.1) using the LFQ-MBR workflow with MaxLFQ minimum ions set at 1 [12]. Additionally, an empirical library was generated through the DIA_SpecLIb_Quant workflow, which utilized the validation tools MSBooster, Percolator, and ProteinProphet followed by input into EasyPQP (v. 0.1.36). The resulting .TSV library output file, filtered at 1% FDR for proteins, peptides, and peptide spectral matches, was used in subsequent single-shot DIA analyses and used to compare the depth of coverage of this library against a predicted spectral library.

To generate the predicted spectral library for DIA analysis, no samples were required, but instead the Uniprot Mus musculus database (UP000005640, 55,315 entries) was uploaded to DIA-NN (v. 1.8.1) [13] with FASTA digest (protease: trypsin/P with one maximum missed cleavage) for predicted library-free search/library generation and deep learning-based spectra, RTs, and IMs prediction activated with the following additional settings: peptide length range of 7 to 30, precursor charge range of 2 to 4, precursor *m*/*z* range of 300–1800, fragment ion *m*/*z* range of 200–1800, and modifications set for N-term Met excision and Cys carbamidomethylation, with variable modifications set to Met oxidation and N-terminal protein acetylation (number of variable modifications = 1).

### 2.5. DIA Mass Spectrometry Analysis

The unfractionated IMG digests were then acquired over 45- and 90-min gradients as assessment samples using DIA-PASEF to determine depth of coverage in a single-shot analysis. The DIA-PASEF method settings included a polygon window consisting of 47 mass steps, each with a mass width of 25 Da and no overlap, an *m*/*z* range of 250–1425, an estimated cycle time of 1.48 s, as well as ion mobility ranges spanning 0.7–1.4 1/K_0_ [V·s/cm^2^] overall. DIA-NN (v. 1.8.1) was used to search these data using both the previously generated empirical spectral library and the predicted library independently with the following settings: both mass accuracy and MS1 accuracy set to 15.0, using isotopologues, and with MBR enabled. Additionally, protein inference was set to genes, single-pass mode was selected as the neural network classifier, robust LC (high precision) was set for the quantification strategy, cross-run normalization was set to RT-dependent, and smart profiling was set for library generation. A 1% FDR cutoff was applied at the precursor level.

In order to assess the differences between the two methods (i.e., comparing the empirical library search to the predicted library search), lists of the unique proteins identified in either the empirical or predicted library approach were obtained from the 90-min DIA analyses (protein group matrixes from DIA-NN). Unique proteins from both the predicted and empirical library approach were uploaded to Ingenuity Pathway Analysis (IPA, Qiagen), where a core analysis was performed to determine which unique pathways were over-represented using a statistical significance cutoff of *p*-value < 0.05 (Fisher’s exact test). Additionally, DIA results for 45-min and 90-min DIA analyses using both empirical and predicted libraries were input into Perseus (v. 2.1.3.0). The proteomic ruler plug-in (v. 1.6.2) [14] was utilized, in which the results were first annotated followed by estimation of copy number using the histone proteomic ruler scaling mode (all default parameters utilized except for averaging mode, where all columns were averaged).

## 3. Results

**Figure 1:**
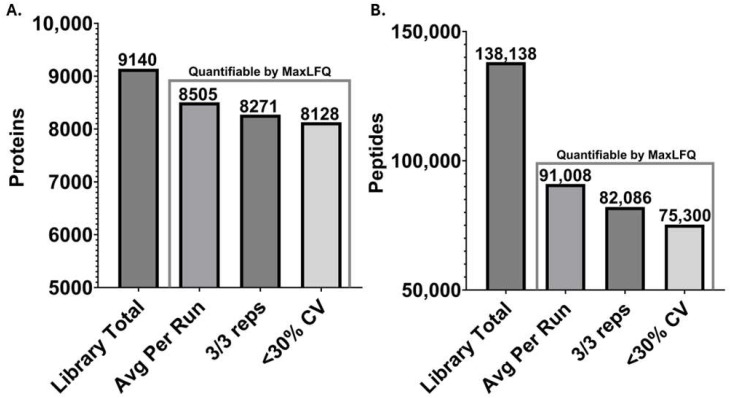
Protein and peptide coverage obtained from the global empirical library search. Number of proteins (**A**) and peptides (**B**) identified during the global search of the files used in the creation of the empirical library for IMG microglial cells. Fractionation consisted of three iST fractions and two IM gas-phase fractions of 0.8–1.05 1/K_0_ and 1.0–1.25 1/K_0_.

**Figure 2:**
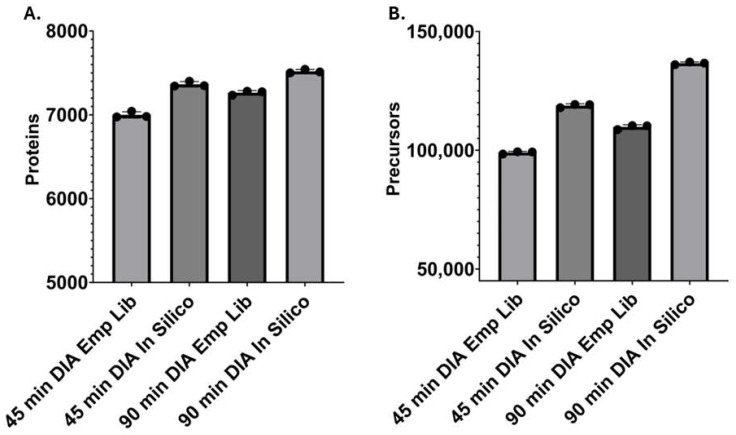
Identification of proteins and precursors from single-shot, DIA analysis of unfractionated IMG cell lysate. Protein (**A**) and precursor (**B**) identification from analyses of unfractionated, iST-processed samples of IMG (*n* = 3). DIA data were obtained using DIA-PASEF with either 45- or 90-min gradient times and searched with either the empirical library or predicted library.

The fractionated IMG cell lysate processed by iST and the peptide fractionation add-on was analyzed by gas-phase fractionation (two ion mobility ranges) using DDA-PASEF in triplicate followed by database searching using MSFragger within FragPipe, resulting in the total identification of 9140 proteins and 138,138 peptides total (Figure 1). Peptide and protein lists from this analysis can be found in the supplemental Excel file (Tables S1 and S2). Across all fractions and replicates, 8505 proteins and 91,008 peptides were quantified on average per run (*n* = 3). These values were obtained with high quantitative precision, with a median CV of 4.5% and average CV of 9.9% observed at the protein level based on MaxLFQ values. Using the empirical spectral library for DIA-NN search, a 45-min gradient single-shot analysis in triplicate averaged 6999 identified proteins per run, and the 90-min gradient DIA searched with the same empirical spectral library identified an average of 7264 proteins (Figure 2). On average, each 90-min DIA analysis identified approximately 79% of the entire empirical library. For DIA-NN searches using the predicted library, 7364 and 7519 proteins were identified on average per run with a 45-min and 90-min gradient, respectively (Figure 2). The predicted library approach accounted for an approximate 3% increase in protein identifications. While the empirical library approach did lead to a small decrease in the identification of proteins, a larger decrease in precursors, approximately 20%, was observed when using the empirical library compared with the predicted library. Overall, both the empirical and predicted library-based DIA workflows allowed for deep proteome coverage in a single shot using either 45- or 90-min gradient methods, identifying approximately 7000 proteins or greater per run. DIA analysis results from DIA-NN obtained from the empirical and predicted libraries at different gradient lengths can be found in the supplemental Excel file (Tables S3–S6).

While comparable numbers of proteins were identified, anticipated differences arose in computational time, with the empirical library taking less time to complete searches. Data were processed on a 3.4 GHz AMD Ryzen 9 5950X with the thread count set to 16 on DIA-NN. For 90-min DIA data (*n* = 3), a predicted library search was completed in 196 min, while a search with the empirical library was completed in 31 min. For 45-min DIA data (*n* = 3), the predicted library completed in 110 min, while the search with the empirical library was accomplished in 19 min. This result corresponds to an approximate 6-fold increase in computational time when using the predicted library approach for both gradient times.

**Figure 3:**
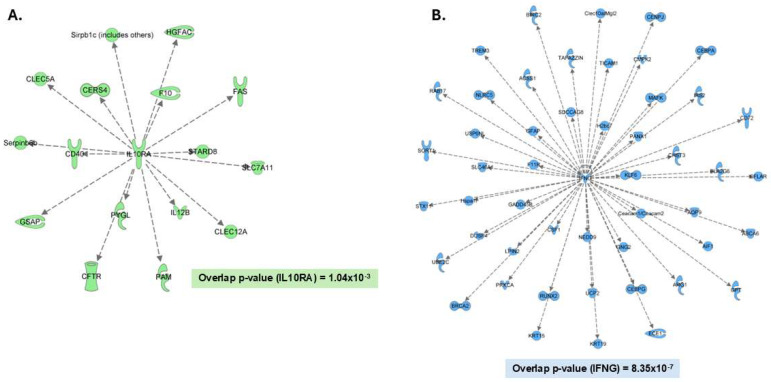
Comparative analysis of proteins identified from predicted library and empirical library-based DIA methods. The predicted library analysis allowed for the identification of 682 unique proteins that were not observed in the empirical library analysis, while 432 were only identified using the empirical library. Dotted lines indicate an indirect relationship between the measured proteins and the predicted upstream regulator. (**A**) Unique proteins from the predicted library analysis identified significant overlap with IL10 receptor subunit A-responsive proteins, while (**B**) the empirical library analysis identified significant overlap with interferon gamma (IFNG)-responsive proteins. Pathways were identified by IPA using its upstream regulator feature (*p*-value < 0.05).

Output from the DIA analysis using the empirical and predicted libraries was further analyzed to determine which proteins were unique to either approach. The majority of the proteins, approximately 7000, overlapped; however, a small but potentially important portion based on biological relevance did not. The predicted library method identified 682 unique proteins, while the empirical library method identified 432 unique proteins that were not in the predicted library dataset. Given the applicability of the empirical library in terms of experimentally relevant precursors related to the DIA single-shot analysis, we observed an additional 5% of the overall proteins that was not identified by the predicted library. The two lists of unique proteins were submitted into IPA independently, allowing us to observe the enrichment of different biological pathways, including those that play critical roles in immune response processes of microglia. For example, the predicted library search by DIA-NN provided more coverage of proteins predicted to be regulated by interleukin-10 receptor subunit alpha (IL10RA) (overlap *p*-value = 1.04 × 10^−3^, 16 proteins) (Figure 3A). On the other hand, the empirical library allowed for the highest overlap of proteins related to the upstream regulator interferon gamma (IFNG) (overlap *p*-value = 8.35 × 10^−7^, 49 proteins) (Figure 3B) as well as proteins related to the upstream regulator lipopolysaccharide (LPS) (overlap *p*-value = 6.66 × 10^−3^, 59 proteins). Therefore, by combining these methods, we were able to increase coverage of pathways related to key microglia regulators and increase the proteome depth of coverage to approximately 8000 proteins.

## 4. Discussion

In this study, we utilized the microglial cell model, the IMG cell line, which has been rigorously characterized based on known microglial functions [9] and shown by our lab to have a high degree of pathway similarities when bioinformatically compared with acutely isolated adult mouse microglia at the proteome level [10]. Therefore, IMG cells represent a more physiologically relevant adult-derived in vitro model for studies of microglia function. Microglia specifically were used to act as a model for testing the applicability of this mass spectrometry-based proteomics methods development to address the proteome complexity underlying this phenotypically diverse cell type.

Upon comparison of the single-shot DIA analysis results using the empirical and predicted libraries, we were able to obtain comparable coverage, with a small increase in protein identifications per run (~3%) using the predicted library; however, further increases in identification (~20%) were observed at the precursor level. Notably, even with modest decreases in protein/precursor coverage, the empirical library results allowed for the unique identification of several immune response pathways relevant to microglial function. It is also notable that our streamlined approach increased experimental throughput with less protein input compared with conventional deep fractionation approaches for proteomic analysis. For example, when compared with a recent study that performed deep proteomic analysis of several mouse and human microglia models, the 10 μg used to create our empirical library was presumably ~10-fold less than the starting material reported in this study and did not require extensive offline HPLC fractionation and subsequent LC-MS analyses [3]. The straightforward peptide fractionation add-on used in our study adapted seamlessly with the iST sample processing and, in combination with considerably faster search times obtained with the empirical library using DIA-NN, resulted in significantly increased experiment throughput for the entire DIA-based workflow. Using the empirical library, we identified 7264 proteins on average per run in a single-shot DIA analysis, which is comparable in coverage to the approximate 7500 average proteins identified per sample for a similar in vitro model, BV2 cells, that was deeply fractionated by separation into 16 fractions and analyzed with multiple LC-MS runs [3]. In a more direct comparison, each replicate of our empirical library detected an average of 8505 proteins with high quantitative precision for analysis, as evidenced by a median CV of less than 5%. By combining both the empirical and predicted library output from the DIA data, collectively, our single-shot proteomic analysis of IMG cells includes approximately 8000 proteins, surpassing that obtained for the deeply fractionated BV2 cells, demonstrating that our simplified method achieves comparable or potentially improved coverage for a single microglial cell model with the TIMS-TOF instrument used in this study. Future work is needed to assess microglial proteome coverage obtained with other next-generation instrumentation such as the newer Orbitrap platforms.

Since the two methods of searching the DIA data with either the predicted or empirical libraries show some differences in identified proteins, our results highlight that these methods can be used in a complementary manner and that the resulting combined output generates a meaningful bioinformatic interpretation of the microglial proteome. Generally, a predicted library approach can be considered as preferable because there are no additional sample input requirements and LC-MS analysis time. Therefore, this approach would presumably contain proteins and pathways relevant to phenotype that may not be represented in an empirical library. However, as observed by the portion of proteins identified by single-shot DIA analysis with our empirical library but not with the predicted library, some proteins highly relevant to microglial function may go undetected through the sole use of a predicted library. For example, these proteins include arginase 1 (ARG1) and cytidine/uridine monophosphate kinase 2 (CMPK2), which are involved in modulating neuroinflammation [15,16]. Both proteins are involved in key immune response pathways regulated by the upstream regulators, LPS/TLR4-mediated signaling and INFG. Conversely, the predicted library allowed for the detection of IL12B, a member of the downstream pathway related to IL10RA and involved in immune regulation, which was not detected using the empirical library [17].

In terms of pathway enrichment, the proteins unique to single-shot DIA analysis using the predicted library method show significant overlap to various downstream pathways, including one regulated by IL10RA, a receptor for the cytokine IL10, which is associated with reduction in the release of proinflammatory cytokines [18]. Both INFG and LPS were identified as upstream regulators related to the unique proteins found solely in the single-shot DIA analysis using the empirical library. INFG is generally considered to be a proinflammatory cytokine that can prime microglia to heighten subsequent responses to proinflammatory insults such as that from the TLR4 ligand, LPS [19]. Potential INFG and TLR4-mediated neuroinflammation, especially over a prolonged period, can lead to neurodegenerative disease [19,20,21]. Due to the important roles of these upstream regulators in immune responses, additional coverage of these pathways is important to capture the full molecular signature of microglial reactivity to various stimuli as well as changes within the local extracellular environment associated with disease pathology. Typically, one would expect a predicted library approach to be able to identify proteins related to unique phenotypes that may not be accounted for in an empirical library, but in this study, we demonstrate that protein coverage unique to the empirical library covers several key pathways relevant to microglial function, which should be a point of consideration when optimizing spectral libraries for single-shot DIA analysis of the microglial proteome.

Given the complementarity of the single-shot DIA results using both libraries, a streamlined empirical library workflow combined with a predicted library search provides an optimized approach for the deepest proteome coverage. This point is particularly important given the limitations of proteomic analysis of microglia, which can include sample amount and overall proteome complexity; therefore, it may be more advantageous in terms of starting protein material to focus on generating additional phenotypes that can be either analyzed separately or pooled to increase library size using our streamlined workflow rather than focusing on deep fractionation. In a recent study describing deep proteomic analysis of human microglial cells (HMC3 cell line), the authors report an average of 4463 proteins per run; however, 10,832 total proteins were collectively identified based at least partially on phenotypic changes induced by a cytokine cocktail treatment [22]. This result highlights the utility of an approach consisting of streamlined fractionation but with the inclusion of multiple microglial phenotypes, representing an optimized experimental compromise that considers proteome coverage closely aligning with the phenotypic diversity of microglia, experimental hands-on time, and the potential limited protein input (more significant with primary microglia or acutely isolated microglia [23]). Additionally, this same method could be applied to in vivo samples as well as other phenotypically diverse cell types such as other immune cells (e.g., dendritic cells, peripheral macrophages, etc.) or vascular smooth muscle cells as examples. Although our methodology was utilized for proteomic analysis of “resting” microglia, further studies are needed to improve microglial proteome coverage by leveraging phenotypic changes that occur via various reactivity stimuli. Altogether, our reported DIA-based workflow has generated an extensive proteome library for deep DIA analysis using a straightforward approach and, to the best of our knowledge, has resulted in the deepest single-shot proteome coverage of microglia reported to date. Comprehensive molecular characterization of microglia at the proteome level will improve insight into microglial reactivity in the context of homeostatic processes of the central nervous system as well as disease conditions such as Alzheimer’s disease, Parkinson’s disease, and amyotrophic lateral sclerosis (ALS) [24].

## Figures and Tables

**Table 1:** Summary of proteomic coverage improvements in rodent-derived microglial cell models. Progressive improvements in the number of microglial proteins identified are listed along with the associated workflows utilized for proteomic analysis.

Source	Microglial Cell Model	Fractionation	HPLC Methods	Instrument	Data Acquisition	Proteins
Bell-Temin et al., 2012 [1]	Rat HAPI cell line (neonatal-derived)	SCX offline HPLC fractionation	90 min gradient, 10 cm column	LTQ Orbitrap XL	DDA	3006
Han et al., 2013 [5]	Mouse BV2 cell line (neonatal-derived)	High-pH RP offline microcolumn fractionation	90 min gradient, 15 cm column	Q Exactive (Orbitrap)	DDA	5494
Rangaraju et al., 2018 [2]	Acutely isolated ex vivo adult-derived mouse microglia	TMT labeling and ERLIC offline fractionation	105 min gradient, 25 cm column	Orbitrap Fusion	(SPS)-MS3	4133
Guergues et al., 2020 [4]	Mouse IMG cell line (adult-derived)	Single shot (no fractionation)	120 min gradient, 75 cm column	Q Exactive Plus (Orbitrap)	Segmented DDA	5062
Lloyd et al., 2024 [3]	Combined mouse ex vivo microglia and BV2 cell line	High-pH RP offline HPLC fractionation	120 min gradient, 50 cm column	Q Exactive Plus (Orbitrap)	DDA and DIA	9633

## Data Availability

All mass spectrometry data have been deposited to the ProteomeXchange Consortium via the PRIDE [25] partner repository with the dataset identifier PXD056436.

## Supplementary Materials

The following supporting information can be downloaded at: https://www.mdpi.com/article/10.3390/proteomes12040035/s1, Table S1: Immortalized mouse microglia (IMG) proteins within the Empirical Library; Table S2: IMG-derived peptides within the Empirical Library; Table S3: Empirical Library search of 45-min IMG DIA data; Table S4: Empirical Library search of 90-min IMG DIA data; Table S5: Predicted library search of 45-min IMG DIA data; Table S6: Predicted library search of 90-min IMG DIA data.
